# The Relationship between Housing and Health: Children at Risk

**DOI:** 10.1289/ehp.7157

**Published:** 2004-08-18

**Authors:** Patrick Breysse, Nick Farr, Warren Galke, Bruce Lanphear, Rebecca Morley, Linda Bergofsky

**Affiliations:** ^1^Department of Environmental Health Sciences, Johns Hopkins University, Baltimore, Maryland, USA; ^2^Housing Consultant, Columbia, Maryland, USA; ^3^National Center for Healthy Housing, Columbia, Maryland, USA; ^4^Cincinnati Children’s Hospital Medical Center, Cincinnati, Ohio, USA; ^5^U.S. Department of Health and Human Services, Washington, DC, USA

**Keywords:** asthma, childhood exposure, environmental toxicants, healthy housing, lead poisoning

## Abstract

In November 2002, the National Center for Healthy Housing convened a 2-day workshop to review the state of knowledge in the field of healthy housing. The workshop, supported with funds from the U.S. Centers for Disease Control and Prevention’s National Center for Injury Prevention and Control and National Center for Environmental Health, was unique in that it focused solely on the effect of housing on children’s health and the translation of research findings into practical activities in home construction, rehabilitation, and maintenance. Participants included experts and practitioners representing the health, housing, and environmental arenas. Presentations by subject-matter experts covered four key areas: asthma, neurotoxicants, injury, and translational research. Panel discussions followed the presentations, which generated robust dialogue on potential future research opportunities and overall policy gaps. Lack of consensus on standard measurements, incomplete understanding about the interaction of home hazards, inadequate research on the effectiveness of interventions, and insufficient political support limit current efforts to achieve healthy housing. However, change is forthcoming and achievable.

Because children spend as much as 80–90% of their time indoors, the possible origins of many of the health risks they face can be traced to homes, schools, and other indoor environments (U.S. EPA 2002). Prevention of environmental disease among children has important social and economic benefits. Landrigan et al. (2002) recently estimated that the total annual costs for environmentally attributable childhood diseases in the United States—lead poisoning, asthma, cancer, and developmental disabilities—is $54.9 billion.

The probable relationship between housing and health inequality, particularly within urban inner-city neighborhoods, has been acknowledged for some time. In 1938, the American Public Health Association (APHA 1938) addressed housing and health in *Basic Principles of Healthful Housing*. In 1971, APHA identified knowledge gaps with respect to housing and health, including the need “to understand and assess better the relative effects on humans of the various stresses that may exist in housing and its environment” (APHA 1971). A renewed appreciation for the housing and health connection led the U.S. Department of Housing and Urban Development (HUD) to implement its Healthy Homes Initiative in 1999.

Measuring the direct impact of housing quality on health was a difficult task in 1938, and this challenge is still with us today. In a recent study of the impact of housing on health, investigators estimated that indices of urban residential quality explained up to 25% of the variability in health status in Japan (Takano and Nakamura 2001). Housing quality remains an important component of health disparities in America and around the world.

The home environment, in particular, represents an important source of fetal and early childhood exposures to biologic, chemical, and physical agents, as well as a strategic opportunity for intervention (Krieger and Higgins 2002). Many studies have linked housing-related factors and health, and we have learned much over the past decades about how to make homes healthier places to live (Saegert et al. 2003). Studies suggest that an integrated approach to housing and health needs to be developed (e.g., Matte and Jacobs 2000).

In November 2002, the National Center for Healthy Housing, a national nonprofit organization dedicated to eliminating residential health hazards to children while preserving affordable housing, convened a 2-day workshop to review the state of knowledge and to help promote the paradigm shift to a healthy housing approach. The workshop was supported with funds from the National Center for Injury Prevention and Control and National Center for Environmental Health of the U.S. Centers for Disease Control and Prevention (CDC). This forum was unique in that it focused solely on housing and the translation of research findings into practical activities to improve health through improved home construction, rehabilitation, and maintenance. The purpose of this article is to present a summary of The Relationship between Housing and Health: Children at Risk Workshop, held 7–8 November 2002 in Annapolis, Maryland.

This workshop had four objectives: *a*) to identify what is known and unknown about the relationship between children’s health and the residential environment; *b*) to identify current “best practices” to address residential health hazards, particularly those that can be readily applied in construction, rehabilitation, and maintenance; *c*) to promote the development of a research agenda concerning residential health hazards and practical housing interventions; and *d*) to identify policy and market options to promote healthy and affordable housing for the nation’s children.

The agenda was divided into four sessions. The first three dealt with housing factors associated with *a*) childhood asthma and other respiratory diseases, *b*) neurodevelopmental and behavioral problems, and *c*) unintentional injuries. The final session focused on how to implement healthy housing programs. During the conference, each session began with two or more invited presentations, a total of 15 presentations for all four sessions, followed by a panel discussion.

This format provided an opportunity for professionals in different topic areas to learn about causes, mechanisms, effects, remediation, and prevention for topics other than their own specialties. It also enabled exploration of possible application of learning from one area to others—identifying commonalities, overarching concepts, and ways to influence policy and guidelines.

The 60 participants included mainly administrators, managers, researchers, and technical experts from major universities and state and federal agencies related to health, housing, and the environment. Participants also included public health professionals, academics, and physicians specializing in allergens, neurotoxicants, pesticides, airborne pollutants, and injury prevention and control. Other participants represented health and housing nongovernmental organizations, state and federal legislative staff, and a few representatives of related industries (pharmaceuticals, construction).

## Residential Determinants of Asthma and Other Respiratory Conditions

### Current state of knowledge.

Asthma, a chronic inflammatory condition of the lung airways, is the most common chronic disease among children today. Although asthma is thought to be caused by one or more mechanisms, there is general agreement that asthma is associated with airway inflammation and hyperresponsiveness. Both environmental and genetic factors are thought to play a role in asthma initiation and exacerbation. In a sensitized individual, small amounts of allergen can result in a large inflammatory response.

It has been estimated that approximately 80% of asthma in children is allergic asthma [Institute of Medicine (IOM) 2000]. Asthma rates are higher in children who are sensitized to allergens (Kattan et al. 1997; Lau et al. 2000; Nelson et al. 1999). Unlike allergic asthma, in which specific immunoglobulin E antibody responses occur, some individuals have nonallergic asthma that is also characterized by inflammation and airway hyper-responsiveness. It is important to note that once asthma has been established, a variety of exposures, including allergens, can trigger an asthma attack or exacerbate symptoms. Asthma often resolves as a child grows up; this happens for about half the children with asthma. However, the person will still have abnormal lung function later in life. In addition, asthma can recur in adulthood. One study suggests that asthma recurs in adults in approximately 20% of the cases (Blair 1997).

Chronic exposure to allergens in the indoor environment from mold, pets, mice and rats, cockroaches, and dust mites is associated with asthma. Indoor moisture sustains mold, pests, dust mites, and bacteria. There appear to be different patterns of sensitization due to varying allergens in different indoor settings. For inner-city home environments, exposure to cockroaches, mice, and rats is also related to asthma and allergic morbidity. Rosenstreich et al. (1997), for example, reported a cockroach sensitization rate of 36% in inner-city asthmatic children.

Indoor air pollutants have also been associated with the development and exacerbation of asthma. Because of cost and other practical limitations, there are relatively few data on personal exposure to common indoor pollutants. One of the most common indoor air pollutants is environmental tobacco smoke (ETS). In a recent review of indoor air quality and asthma, the IOM concluded that there is sufficient evidence to associate ETS exposure with the development and exacerbation of asthma (IOM 2000). According the IOM review panel, there is suggestive evidence that nitrogen dioxide can exacerbate asthma, but there is inadequate evidence to support an association with the development of asthma (IOM 2000). The volatile organic compound formaldehyde has received research and public attention, but its role in the development or exacerbation of asthma is not clear.

Three outdoor pollutants—ozone, sulfur dioxide, and fine particles—are also known to exacerbate asthma. Indoors, SO_2_ and O_3_ are readily adsorbed onto surfaces and taken out of the air. Thus, there seems to be less of an indoor problem with these chemicals. The relationship between in-home particulate matter exposure and asthma is not well researched. Additional research is also needed on the combined effects of indoor and outdoor pollutants.

### Best practices to address housing-related asthma risk factors.

Because exposure to allergens has been identified as a major source of airway inflammation, asthma control efforts have focused on allergy avoidance. Accordingly, the basic strategy to alleviate respiratory symptoms is to determine to which allergens a person is sensitive and then follow a set of steps to avoid those specific allergens. Because half of asthmatics have multiple (three or more) sensitivities (Eggleston 2000; Huss et al. 2001), several actions may have to be undertaken over long periods of time to control their symptoms.

Efforts to control asthma require consistent application of various measures. Practical issues, including the cost of equipment and the time involved to keep a home sufficiently clean, can affect the patient’s ability to carry out recommended steps. Factors affecting behavior change need to be understood and incorporated into the design of interventions. Interventions should be promoted that are most likely to give positive results (i.e., reduced attacks, for example) and encourage continued compliance. Examples include the Master Home Environmentalist program (MHEP)—a community-based program that focuses on indoor sources of pollution, pesticides, and moisture—as well as programs aimed at helping builders construct healthy homes, such as that of the American Lung Association and the Asthma Regional Council. The MHEP is operated by the American Lung Association of Washington State and has spread to four other states. Trainees can use the Home Environmental Assessment List questionnaire to assess the home environment and negotiate an action plan with the family (Leung et al. 1997).

### Knowledge gaps and research needs.

The complex mixture of allergens in the home setting presents several challenges. There could be interaction effects among allergens to cause sensitization and attacks and worsen the condition. Presently, there is no indicator of total allergen burden, and if it is developed, it would have to correlate well with asthma development and exacerbation.

Within the home, there are several measurement issues related to allergens. For example, the relationship between a surface allergen sample and the inhaled dose is not known. Additionally, the location of sample measurements can give different levels, so there is need to give attention to sample site selection.

Other research needs include the following: *a*) Interactions and synergy among allergens, mixtures, and multiple risk factors are not well understood or documented. *b*) The dose–response relationship between mold and the development of specific disease states is not adequately documented. *c*) Additional research is needed to document the optimal and feasible behavior change strategies required to maintain respiratory health and improve compliance with proven interventions. *d*) The relative benefits of various interventions such as rehabilitation and renovation versus intensive cleaning have not been measured.

## Environmental Neurotoxicants in the Home

### Current state of knowledge.

The most significant neurotoxicants found in residential settings are lead, pesticides, and ETS (Jordaan et al. 1999; Lanphear et al. 2000; Whyatt et al. 2002). Lead toxicity affects the brain and neurodevelopmental processes, and its effects are irreversible. Lead paint dust is the primary source of exposure in homes, rather than pieces of lead-based paint or lead in soil. In the United States, 17% of low-income children have blood lead levels > 10 μg/dL, the level that is considered elevated by the CDC. Recent research suggests that there is probably no lower-level threshold of blood lead (Bellinger and Needleman 2003; Canfield et al. 2003). The biologic mechanisms involved in lead poisoning are not yet well understood (Silbergeld 1992), although it is known that lead disrupts processes regulated by calcium and changes synapse formation (Bressler et al. 1999). Even at quite low levels (2.5–10 μg), deleterious effects of lead can be detected (Canfield et al. 2003). An inverse relationship between blood lead concentration and arithmetic and reading scores was observed for children with blood lead concentrations < 5 μg/dL (Lanphear et al. 2000).

Smoking cigarettes during pregnancy has effects on fetal development and the baby’s health and development after birth. Estimates of women who smoke during pregnancy range from 25 to 44%. A mother’s smoking habits are closely associated with a child’s exposure, probably because the child is with the mother for more time and in closer proximity. Children’s blood cotinine levels have been shown to increase with increasing numbers of smokers in the household (Jordaan et al. 1999).

Exposure to tobacco smoke during and after pregnancy is associated with prematurity, low birth weight, low Apgar scores, poor growth of infants, and dysfunctional behavior (Bauman et al. 1991; Eskenazi and Trupin 1995; Fergusson et al. 1993; Williams et al. 1998). Currently, evidence related to child development and behavior is stronger for prenatal than for postnatal exposure. Recent research using blood cotinine levels as an indicator of exposure to ETS shows a robust inverse relationship between postnatal cotinine levels and cognitive scores (math and reading) in 6- to 16-year-old children. The relationship remains statistically significant after controlling for various characteristics (Lanphear et al. 2000).

The mode of action of most pesticides is to be neurotoxic to pests. It is reasonable to assume, therefore, that they will also have neurotoxic effects on humans. There is a growing body of evidence suggesting that public exposure to cholinesterase-inhibiting pesticides (organophosphates and carbamates) is a health concern (Whyatt et al. 2002). The impact of organophosphate and carbamate exposure on children has not been extensively researched, particularly with respect to neuro-behavioral testing.

In inner-city home environments, indoor exposures to some pesticide toxicants can be frequent and at high levels due to cockroach and rodent problems. Whyatt et al. (2002) recently reported on the pesticide use of inner-city residents in New York City. This study documented widespread pesticide use, and in the case of diazinon, the exposure for some women may have exceeded healthy levels based on the U.S. Environmental Protection Agency (EPA) reference dose. Eighty-four percent of the women questioned as a part of this study reported that pest control measures were used in the home during pregnancy. Not surprisingly, a number of organophosphate (both chlorpyrifos and diazinon) and carbamate (propoxur) pesticides were detected in air samples, maternal blood, and cord blood samples (Perera et al. 2003).

### Best practices to address exposure to housing-related neurotoxicants.

The steps needed to prevent childhood exposures to neurotoxicants are founded in core public health practice. They include identifying sources of exposure, defining unacceptable levels of exposure, developing and testing interventions, and, finally, implementing effective policies and screening programs. Intervention strategies include (in increasing order of effectiveness and cost) education, enforcement, and engineering controls with an emphasis on primary prevention. It is difficult to address detrimental effects on neurodevelopment when children are exposed to multiple neurotoxicants at the same time. It is common for inner-city children to be exposed to lead, ETS, and pesticides both prenatally and postnatally. To be effective, intervention efforts should address these multiple exposures at the same time.

In the case of childhood lead exposure, there is an extensive body of literature documenting the impact that various methods of lead hazard control have on dust and blood lead levels (Galke et al. 2001; Haynes et al. 2002; Niemuth et al. 1998; Staes and Rinehart 1995). Niemuth et al. (1998) summarized the literature from 1980 through 1998 and included both trials and observational studies. Generally, the studies report successful reductions in dust lead levels and in blood lead levels when initially > 20 μg/dL. To date, the published data on the effectiveness of specific lead hazard control treatments have been too limited to draw conclusions about the relative effectiveness of specific lead hazard control approaches (e.g., window replacement, paint stabilization).

Although pesticide use in the home is common, designing and implementing intervention strategies are difficult because of the lack of basic toxicity testing information specific to neurodevelopmental effects. Without adequate toxicity and human exposure data, it is not possible to target control strategies. Basic toxicity testing for neurodevelopmental effects as well as aftermarket health and exposure surveillance should be mandated. Further, we need to shift programs away from screening of children to screening of homes. This will require the development of health-based screening guidelines similar to those developed for lead.

Besides the elimination of ETS, three methods of control—air filtration, ventilation, and smoke containment—could be used to reduce the presence of tobacco smoke in the home environment. Although it is clear that eliminating smoking (either by quitting or smoking outside and away from children) will reduce ETS exposure (Johansson et al. 2004; Wakefield et al. 2000), there is little research on the efficacy of active engineering control methods (i.e., ventilation and/or filtration in the home). A controlled pilot study of high-efficiency particulate air-carbon potassium zeolite filters has shown that they are able to reduce nicotine in the air (Aligne CA et al., unpublished data). Unintended negative consequences of interventions must be considered. For example, it is not clear whether air purifiers that generate O_3_ present a health problem that might mitigate their effectiveness. Nonetheless, education and smoking cessation programs are the most commonly used interventions for ETS exposure. Smokers must be educated about the impact of ETS on children and the need to avoid smoking in their presence. In addition, educational efforts should continue to target pregnant mothers to prevent prenatal exposure. Further investigation is also needed to understand why some families take steps to reduce ETS exposure and others do not.

### Knowledge gaps and research needs.

Although there may be limitations, laboratory studies are needed to increase knowledge about the active toxicants found in complex mixtures present in the home environment, such as house dust and ETS. Basic laboratory studies can provide early indications about the likely effects of environmental toxicants such as pesticides and ETS because they are typically cheaper and faster than epidemiology studies. However, issues inherent in studying animal models and extrapolating to human beings underscore the need for epidemiology studies.

Consistent outcome measures across studies, such as what to measure and agreed-upon cutoff points, are needed. Also, interactions and synergistic effects of multiple toxicant exposures on neurodevelopment need additional research. Finally, premarket neurotoxicity testing of home pesticides and postmarket surveillance will help fill knowledge gaps.

Several measurement considerations were also raised during the workshop. These included appropriateness of measures of exposure and dose, ability to detect differences and interpret them, and comparability across studies. Scientific agreement on the best measures of specific exposures and effects would make it easier to compare results of different studies and to perform meta-analyses. Precise measurement of actual exposures in the home environment would improve the ability to find effects and draw clear conclusions. Additionally, there is a need to develop new and less costly techniques to measure and analyze pesticide exposures.

There is insufficient evidence that educational programs and other interventions about ETS and pesticides result in less exposure by pregnant women and after a child is born. Besides reported behavior, additional measures of the behavior effects of toxicants need to be developed.

## Unintentional Injury of Children in the Home

### Current state of knowledge.

Between 1985 and 1997, home injuries accounted for almost two-thirds of all fatal unintentional injuries occurring to U.S. children and adolescents, with mean residential death rates for children and adolescents varying markedly by age, race, and geographic location.

Data from the National Hospital Ambulatory Medical Care Survey for 1993–1999 [National Center for Health Statistics (NCHS) 1993–1999] for children < 20 years of age show that injuries accounted for 11 million visits to the emergency department (ED). Injuries occurring in the home accounted for four million of these visits. Children in the youngest age groups had significantly higher ED visit rates, similar to rates for fatal injuries. Residential injuries leading to ED visits were highest for children 1–4 years of age. In addition, males had higher ED visit rates than did females (Phelan et al., in press). For children < 1 year of age, 93.5% of all deaths due to injury occurred in the home. That proportion declined progressively with age through adolescence, falling to 38% for 15- to 19-year-olds (Nagaraja J et al., unpublished data; Pollock et al. 1988). Similar results were found in a recent study reporting that 80–90% of injury deaths among children < 5 years of age occur in the home, compared with 80% for 5- to 9-year-olds and 60% for 10- to 14-year-olds (Lanphear B et al., unpublished data).

The fatality rate for residential injuries has declined by about 25% since 1985. Despite the decline, residential injury death rates are substantially higher for African-American children than for other race groups. Unlike injury death rates, nonfatal residential injury rates by race were similar (Nagaraja J et al., unpublished data).

Injuries of differing severity may be associated with different risk factors. The different injury severity outcomes must be examined separately to define the risk factors that describe the linkage between children’s homes and the injuries they might suffer.

Falls are the leading type of residential injury for children; they account for an estimated 3 million visits to the ED. The primary residential hazards associated with falls are lack of safety devices such as grab bars, safety gates, or window guards; structural defects in the home; and insufficient lighting on stairs and other areas (Battelle Memorial Institute 2001). Risk of death from fire is higher in the South and Southeast than in other regions of the United States, and children < 5 years of age and the elderly are at higher risk than other age groups. Scalds, nonfire burns, and poisoning injuries occur among children fairly often. Burns account for about 185,000 ED visits annually for children < 20 years of age (Phelan et al., in press), and 95% occur among children < 5 years of age (CDC 2002). Infants and toddlers are at higher risk of accidental poisoning requiring an ED visit than are children 5–19 years of age (Phelan et al., in press).

Although large national sources of injury data provide some key insights into injury rates, there is a need to improve injury epidemiology and surveillance to collect data on injuries that do not result in hospital visits. In addition, better data are needed to evaluate the determinants of injury rate variability in order to design effective intervention strategies.

### Best practices to address housing-related injury risk factors.

The most important home safety actions documented in the literature are reduction of the temperature of hot water heaters to 120°F to prevent scald burns; use of stair fences; installation of window guards, especially in high-rise buildings and upper stories of homes and apartment buildings; installation and maintenance of smoke alarms; installation and use of cabinet locks; and segregation and locking away of poisons.

Smoke alarms are a key means to prevent injury or death due to home fires. When homes have functioning smoke alarms, there is a 50–80% reduction in injury and death due to residential fires (CDC 2002). A pilot program for comprehensive residential fire prevention showed promising results (Jackson 2002). Components of the program included installation of smoke alarms, educational activities, and cooperation among local health departments, fire departments, community organizations, and the media. The smoke alarm program found that 85% of alarms were operational when follow-up was done. This program approach might be a useful model for prevention of other types of residential injuries, but it should be evaluated for effectiveness.

### Knowledge gaps and research needs.

There is a need for better data on residential injuries of children and for meaningful measures of injury outcomes. Questions on injuries and injury prevention might be included in household surveys to better capture injuries that do not result in hospital ED visits. Overall surveillance of home injuries must be improved, as well as the capability to identify, measure, and report on new and emerging home hazards. Higher-quality information should be obtained from existing sources of home injury data, such as fire departments and fire marshals, to facilitate development of prevention activities.

Behavior change is a challenging aspect of injury prevention. Researchers and policy-makers need to understand what motivates and enables parents to take injury prevention measures in the home and sustain such behavior over time. Concerted efforts should also be made to tap the vested social and economic interests of employers and insurers in reducing injuries.

## Translating Healthy Homes Research into Action

Understanding the relationships between residential environmental hazards and children’s health problems is a necessary precedent to preventing those problems. Equally important is an understanding of how to translate that knowledge into preventive action by developing and promoting the most cost-effective techniques to assess and control hazards. To that end, workshop participants suggested several action steps to facilitate the pursuit and dissemination of translational research.

### Increasing funding for research and evaluation of demonstration projects to determine how best to assess and control hazards.

At present, the HUD Healthy Homes Initiative is the predominant source of funding dedicated to understanding how to prevent diseases and injuries associated with housing hazards. Because of the small appropriation and the structure of the program, HUD funds only 2-year projects, which makes it challenging to develop definitive conclusions. Congress should provide HUD with a longer-term authorization and mission, and it should direct other agencies such as the U.S. EPA, CDC, and National Institute of Environmental Health Sciences to fund long-term studies, including randomized control studies. Those studies should be developed and managed by collaborations that include medical and public health schools, research-oriented housing organizations, and members of those communities most affected by the issues.

Additional long-term funding will be available only if the interested scientific and advocacy communities make a major effort to provide the potential funders and key legislators with existing evidence that diseases can be prevented and money saved in the long run by supporting substantial expansion of the healthy housing effort.

### Enforcing existing hazard elimination and control regulations and considering enacting new regulations.

Federal, state, and local legislation, regulations, and guidelines already exist that could materially reduce residential hazards. The existing comprehensive lead laws, regulations, and guidelines set the example. Most housing codes aim to prevent excess moisture intrusion and pest infestation and require ventilation. Just as code requirements for smoke alarms and stair and window guards reduce unintentional injuries, effective enforcement of those codes could sharply reduce such allergens as cockroaches, mold, and rat and mouse dander.

For the most part, these requirements and their enforcement are aimed at individual hazards. Relatively minor changes in the codes could increase their effectiveness across hazards. Focused cross training of sanitary and building code inspectors using a common assessment protocol could increase efficiency and thoroughness in identifying hazards as well as help reinforce any educational program. A common assessment tool would also enable researchers to compare assessment results with any health conditions reported.

Additionally, government agencies should promote basic healthier housing construction standards for housing receiving government funding. The government should evaluate the cost and effectiveness of these standards in increasing durability, decreasing maintenance and energy costs, and lowering costs associated with children’s exposure to hazards. The results of the research and evaluations described above should be completed and the results disseminated widely to build a consensus and affect policy change. For example, government agencies and nongovernmental organizations could sponsor conferences to enable medical organizations, congressional staff, and foundation representatives to collaborate and discuss the results of research and evaluations.

### Pursuing market-based approaches to eliminate or control hazards.

Ideally, the market consisting of informed homebuyers and renters would induce landlords, homebuilders, renovators, and remodelers to make housing safe and healthy. However, in some cases current buyers and renters value aesthetics and lower costs over health issues that they do not understand, and builders are unlikely to change their plans voluntarily, particularly if the change adds even marginally to costs. In many cases, individuals with limited means are forced to choose between healthy housing and affordable housing and are therefore not in the position to influence market forces.

Public education programs featuring the dangers of unsafe and unhealthy housing should be mounted to stimulate demand for healthy market rate housing. This is especially true in low-income housing where the stakeholders (i.e., tenants) lack the political capital needed to stimulate change.

Builders are motivated primarily by decreasing the time and lowering the cost required for construction. It can and should be possible to demonstrate that moisture-resistant housing can be built for the same price and within the same time frame as housing that is constructed otherwise. Moreover, healthy housing is likely to reduce buyer complaints and lawsuits. Experimental programs to train New England builders that simple moisture resistance techniques are practical and inexpensive were well received (Tohn 2002). Demonstrating that safe and healthy housing may mean lower maintenance costs and healthier residents should motivate owners, housing finance agencies, and even banks to include healthy housing techniques in their specifications and underwriting standards.

Fear of liability induced property owners to accept lead safety standards. Publicity about illness apparently caused by mold has threatened the availability of insurance in parts of the country. So far, few if any lawsuits have been brought against rental property owners for exposure to allergens or pollutants, presumably because control of those hazards is considered to be the resident’s responsibility. As the relationship between structural conditions that cause excess moisture and disease becomes clearer, such lawsuits may become more common. Insurers and lenders could include moisture resistance measures in their underwriting guidelines. Additionally, insurers could consider rate discounts for properties that follow housing guidelines.

### Persuading medical and policy organizations that eliminating or controlling hazards in housing should be given high priority.

The medical establishment naturally focuses on treating diseases such as asthma, lead poisoning, and cancer with drugs rather than eliminating or reducing exposure to the hazards that contribute to the diseases. Because the hazard of lead in household dust is so clearly the primary cause of childhood lead poisoning, the medical community has shifted its emphasis to a primary prevention approach as opposed to treatment. Asthma and cancer are much more complex diseases, and the relationship between disease and allergens, pests, pollutants, pesticide residue, and other chemical and biologic hazards in housing is less clearly understood. Primary prevention approaches to a wide spectrum of housing hazards need to be both developed and implemented. Brunekreef et al. (1989) suggest that failure to control dampness, mold, and moisture in homes and the associated pests, bacteria, and dust mites has the same large impact on children’s health as does ETS.

Research proposed in this workshop summary is critical to convincing the medical profession, Congress, government agencies, foundations, and other stakeholders that they should also focus on housing if these diseases are to be prevented. Because research and demonstrations show that assessing and treating hazards in housing can reduce the incidence and severity of disease, a concerted effort must be mounted to disseminate the data and conclusions to medical professionals, policymakers, and funders.

## Conclusions

Four major themes emerged from the expert presentations and panel discussions: *a*) Although all of the mechanisms are not yet well studied and described, the built environment, including residential housing, is an agent of health (or illness) for children. *b*) The body of research around lead toxicity can serve as a model for analysis and exploration for other environmental hazards. *c*) Studies that can establish linkages among the residential environment, children’s health status, and interventions face ethical and practical constraints, which may limit the range of options available. *d*) Social determinants influence who is at risk for exposure or injury, how they react to those substances or risk factors, and the efficacy of interventions.

Participants identified four global research gaps and policy issues requiring further consideration and resources: *a*) Home hazard measurement techniques and standards have not been developed for all hazards. Until better measurements of the direct impact of housing quality on health are developed, the relationship will be undervalued. *b*) Translational research is lacking. The efficacy of interventions has not been sufficiently demonstrated through rigorous, long-term studies. *c*) Interactions and synergies among hazards are presumed to exist but are not well documented or understood. *d*) A broader coalition of researchers, policy-makers, appropriators, and advocates must be engaged to fill data gaps, support needed research, and pursue policy change.
